# A review of data on laboratory colonies of bed bugs (Cimicidae), an insect of emerging medical relevance

**DOI:** 10.1051/parasite/2015021

**Published:** 2015-06-19

**Authors:** Arnaud Cannet, Mohammad Akhoundi, Jean-Michel Berenger, Gregory Michel, Pierre Marty, Pascal Delaunay

**Affiliations:** 1 Inserm U1065, Centre Méditerranéen de Médecine Moléculaire, C3 M, Université de Nice-Sophia Antipolis 151, Route St Antoine de Ginestière BP 2 3194 06204 Nice Cedex France; 2 Service de Parasitologie-Mycologie, Hôpital de l’Archet, Centre Hospitalier Universitaire de Nice 06202 Nice France; 3 URMITE, UM63, CNRS 7278, IRD 198, Inserm 1095, Faculté de Médecine, Université d’Aix-Marseille 13385 Marseille France

**Keywords:** Bed bug, Physical factors, Physiological factors, Laboratory colonies

## Abstract

Cimicidae are hematophagous Heteroptera, feeding on human blood, that have been the subject of significant medical investigation. In particular, they have been colonized under laboratory conditions to study their medical relevance. Laboratory colonization of these bugs is a multifactorial phenomenon. Our goal was to conduct a comparative literature review to classify the published data, demonstrating preferred bed bug colony conditions. We show that physical factors including temperature, relative humidity and photoperiod, and physiological factors such as type and frequency of blood meals play important roles in laboratory colonies. Any change in these factors produces changes in life-cycle duration. Temperature and blood meal are the most important factors, with a marked impact on the life-cycle of laboratory populations, depending on the species. A wide range of temperatures (15–34 °C) and relative humidity (46–75%) with an average of 25 °C and 59% were found for these colonies. Two widely used blood sources for the colonies were rabbits and humans.

## Abstract (fr)

Les Cimicidae sont des Hétéroptères hématophages se nourrissant de sang humain, qui ont fait l’objet de nombreux travaux médicaux. En particulier, ils ont été établis en colonies dans des conditions de laboratoire pour étudier leur intérêt médical. L’établissement de colonies de punaises de lit est un phénomène multifactoriel. Notre objectif était de procéder à un examen de la littérature comparée et de classer les données publiées soulignant les conditions de colonie préférées des punaises. Nous montrons que les facteurs physiques (température, humidité relative, photopériode) et physiologiques (type, fréquence des repas de sang) jouent des rôles importants dans les colonies de laboratoire. Toute modification de ces facteurs produit des modifications des durées des cycles de vie. La température et le repas sanguin sont les facteurs les plus importants, ayant un impact marqué sur le cycle de vie de populations de laboratoire, selon les espèces. De larges gammes de température (15 à 34 °C) et d’humidité relative (46 à 75 %), avec des moyennes de 25 °C et 59 %, ont été signalées pour ces colonies. Deux sources de sang largement pour ces colonies étaient les lapins et les humains.

## Introduction

**Figure 1.**
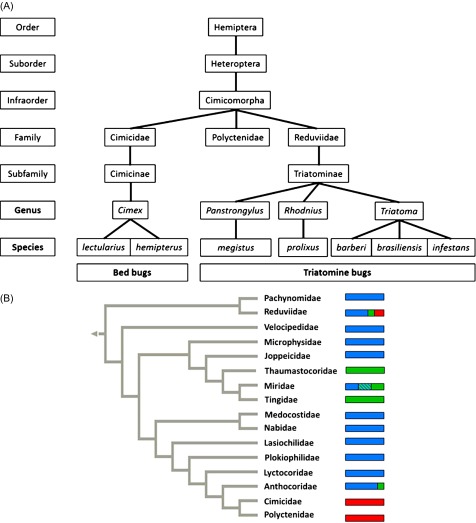
(A) Classification of the Hemiptera order with a focus on genera of medical relevance, (B) Cladogram of Cimicomorpha showing feeding habits (adapted from Schuh & Stys, 1991 [32]).

Hematophagous insects represent a minority of the broad class Insecta and have significant impact on human health. They include the insects of the Hemiptera order and the infraorder of Cimicomorpha (16 families) in which three families, Cimicidae, Reduviidae (only Triatominae) and Polyctenidae, are hematophagous at all stages and are medically relevant [32, 34] (Fig. 1). Cimicomorpha has wide feeding diversity, mostly phytophagous or entomophagous, and only three of the families mentioned above have a blood diet.

Cimicidae are important ectoparasites for human health and more broadly for homeothermic or heterothermic vertebrates [10]. The Polyctenidae family is not considered here.

Several investigations have been performed on various biological, ecological and vectorial aspects of hematophagous bugs in the past decades. Understanding these pests or vectors including their behaviour, environmental parameters, and dynamics of disease, is essential to better clarify disease circulation and transmission by these insects to devise control strategies.

Triatomine bugs are already known to be the vectors of *Trypanosoma cruzi*, the etiological agent of Chagas disease. According to the recent experimental study carried out by Salazar et al. (2014) [30], bed bugs seem to be a competent vector of *T. cruzi*, although their natural epidemiological role is unclear. Given the global distribution of bed bugs, this will be an important medical issue, leading to difficulties in control strategies.

As a result, laboratory rearing of bed bugs is particularly important in understanding their vectorial role and in studying the association between natural conditions and laboratory situations for further experiments. Previous studies have demonstrated the adaptability of bugs to a wide range of environmental factors [2]. Laboratory colonies should experimentally reproduce the natural living conditions of bugs in a controlled environment to obtain consistent results in scientific research.

Unfortunately, detailed information on laboratory conditions was sometimes not available and the roles of some environmental parameters, such as physical or physiological factors, for these insect colonies were studied although they were rarely comparable. Moreover, most studies were carried out on certain species without consideration or comparison of multiple environmental factors together with natural conditions.

It will be necessary to find a balance between the natural environment and laboratory conditions for rearing of bugs over the long term. Therefore, it would be logical to have laboratory bug colony conditions closely related to the situations surrounding human environments to investigate their biology, focusing on medical issues.

On the basis of published data, coupled with results of studies conducted by senior researchers, we examined and compared environmental factors in laboratory conditions for bed bugs. Our goal was to assess different physical factors, i.e. temperature, relative humidity (RH) and photoperiod, and physiological factors including type and frequency of blood meal, and life-cycle, that play important roles in the complete life-cycle of laboratory bug colonies.

## Focus of this review

Bed bugs have been spreading worldwide since the 1990s and mainly in developed countries. These species have high ectoparasitic evolution due to a lifestyle close to their hosts. The Cimicidae is a cosmopolitan family comprising 24 genera and 110 known species. Birds or bats are the primary hosts for *Cimex columbarius*, *Cimex pipistrelli*, *Cimex insuetus*, *Oeciacus vicarious*, *O. hirundinis*, *Stricticimex parvus* and *Leptocimex boueti* or primitive hosts for *Cimex lectularius* and *Cimex hemipterus* [33]. All these species also feed on humans [8, 31, 35]. This review focuses on *C. lectularius* (cosmopolitan species) and *C. hemipterus* (tropical species), two well-known species mainly living in human habitats.

## Literature review

Based on review of the literature including entomological publications, we propose an update of laboratory conditions and breeding methods for bed bugs. To describe the complete life-cycle of laboratory colonies, we retained the main physical (temperature, RH and photoperiod) and physiological (type and frequency of blood meal, and life-cycle) factors that were studied for these insects, summarized in Table 1.

**Table 1.** Laboratory records of bed bugs according to the literature.

Species	Rabbit		Human		Rodent		Chicken		Horse		References
	°C (RH)	LC	°C (RH)	LC	°C (RH)	LC	°C (RH)	LC	°C (RH)	LC	
*C. lectularius*											
	15 (–)	118.86^a^	18.8 ± 0.1 (46.2 ± 1.0)	_^b^	18.8 ± 0.1 (46.2 ± 1.0)	_	22–25 °C (–)	35	28 (–)	25.3^a^	[1, 12, 18, 26]
	18 (–)	65.58^a^	27 ± 0.5 L (50 ± 5)	35^b^	28 (–)	23.2^a^	26.1–26.5 (68.90)	_^b^	28 (–)	20.8^b^,^c^	[3, 12, 19]
	23 (–)	28.96^a^	27 ± 0.5 F (50 ± 5)	33^b^	_	21.6^a^					[3, 12, 19]
	25 (–)	23.96^a^	28 (–)	21.1^a^							[12, 19]
	26	42^b^	28 (–)	24.4^a^,^b^							[11, 12]
	27 ± 0.5 L (50 ± 5)	37^b^									
	27 ± 0.5 F (50 ± 5)	52^b^									[3]
	28 (–)	14.07^a^									[3]
	28 (–)	20.8^a^									[19]
	28 (–)	23.8^a^,^b^									[12]
	28–32 (75)	36.9 ± 8.2									[12]
	34.5 (–)	15.28^a^									[3]
*C. hemipterus*											
	28–32 (75)	39.9 ± 7.0	26 ± 2 (70 ± 5)	24.47–24.62^a^							[16, 17]
			26 ± 2 (70 ± 5)	17–20^a^							[16, 17]

a without egg,

b artificial feeding,

c without egg and instar V, °C: temperature, RH: relative humidity (%), LC: life-cycle, L: laboratory, F: field.

## Physical factors

*Temperature* – Bloodsucking bugs live naturally in a wide range of environments. Cimicidae were originally a wild species that have in part adapted to ecological niches created by human activities. Environmental factors can influence bug distribution in their natural habitat [21].

Temperature can strongly impact the development and behaviour of these insects. Several studies focused on the influence of temperature on this species (Table 1).

A wide temperature range (15–34.5 °C) with an average of 25.5 °C was described for bed bug colonies; 32–33 °C and 28–29 °C were the most favourable temperature ranges for *C. lectularius* and *C. hemipterus* [24] with higher temperatures of 35–46 °C decreasing bed bug survival time [17]. *C. lectularius* proved extraordinarily resistant to desiccation [4, 5]. *C. hemipterus* was more tolerant of temperature variations, e.g. 40 min at −20 °C, <7 days at 0 °C [25]. Temperatures of 45 °C and 39 °C for 1 h have been reported to be lethal limits for *C. lectularius*. These bugs did not move spontaneously when exposed to temperatures < 9 °C. Nymphs were sensitive to low temperatures that influenced blood digestion duration lasting 1–2 days at 23 °C and 6 days at 15 °C. Similarly, egg hatching varied from 3–14 days at 23 °C to 22–52 days at 14 °C. No hatching was observed at temperatures > 37 °C or < 13 °C [19].

Moreover, based on our calculations using Johnson’s results [19], increasing temperature from 15 °C to 28 °C or from 18 °C to 23 °C accelerated egg-adult time by 744% and 126%, respectively. Conversely, increasing temperature from 28 °C to 34.5 °C reduced this effect (−8%) on bed bug colonies [19].

*Relative humidity* – RH is the second physical environmental factor that plays an important role in the biology of these insects, although it is less important than temperature [28]. The reported effects of RH on bug colonies were highly variable between studies and ranged from no impact to significant effects.

High RH tolerance (10–70%) enables bed bugs to withstand desiccation, particularly after a blood meal [33]. The contribution of heat shock proteins to their water-retention strategy, combined with aggregation behaviour, could explain the extreme resistance of *C. lectularius* to desiccation [4, 5]. While RH has no effect on the egg stage duration or between feeding and molting times, 75–90% RH is the value most favourable for successful bed bug hatching. Also, 70% RH is optimal for a temperature range of 16.4–34.4 °C [19]. High temperatures surpassed the RH effect on bed bug survival [16]. Low RH had a harmful effect by shortening survival [24], e.g. 33% RH significantly decreased survival of adult *C. lectularius* compared to 50–75% RH. Bed bugs are perfectly adapted to human habitats at 22–24 °C and 30–50% RH [4, 5].

*Photoperiod* – This factor was not indicated in most publications. According to the literature, laboratories maintained light/dark cycles between 12 h/12 h and 16 h/8 h for bed bug colonies.

## Physiological factors

*Type of blood meal* – Many bloodsucking insects prefer particular groups or species of hosts with varying specificity. Some are host-species specific but others accept feeding on a wide range of hosts.

This feature is also seen in hematophagous Heteroptera. Most Heteroptera use a piercing-sucking mouth apparatus with a morphological adaptation for aspirating vegetable fluid or insect body fluids but some have been evolved to blood feeding. A blood meal is required to moult to the next stage and is essential for egg laying and development.

Hematophagous Heteroptera are found in a wide variety of habitats and each species has a predilection for a specific microclimate. This adaptation is not only for physical conditions but also for physiological factors to find a suitable host.

These bugs feed on a wide range of animal hosts during their life-cycle. Host accessibility is a major factor shaping bug blood-foraging patterns.

Several experimental studies have shown that the fecundity of a bloodsucking insect depends on the host on which the insect feeds. Fecundity can be reduced by a slower insect development rate, shortened longevity, skewed sex ratio, less food intake or slower digestion [20].

For bed bugs, authors used live birds, mammals or an artificial feeder with different blood sources, while rabbit and human blood was the most widely used (Table 1). *C. lectularius* and *C. hemipterus* are natural ectoparasites of birds and bats. In their absence, human blood is the preferred food [33]. The Cimicidae have relatively few natural host choices, unlike Triatominae.

According to the literature, bug colonies were fed in two ways, either live animals or artificial feeding with different blood sources. There were also two approaches for artificial feeding. The first approach was to feed the bugs with the same or similar animals to their natural hosts. The second feeding strategy used different animals due to accessibility and ease of use.

In live animal-fed colonies different blood sources were used. Rabbit and human blood was the most commonly used source for bed bug colonies (Table 1). Montes et al. (2002) [23] devised the so-called water bath feeding system to feed the bed bugs that adequately replaced a living animal used for laboratory colonies since the first description.

Chin-Heady et al. (2013) [11], and Aak and Rukke (2014) [1] designed two new simple artificial feeders as an alternative to maintain laboratory bug populations at lower cost.

Barbarin et al. (2013) [3] used artificial feeding with a Parafilm^®^ membrane for two *C. lectularius* populations from field and laboratory colonies. The life-cycle of laboratory populations fed human blood was 32% shorter than bed bugs fed rabbit blood and only 11% shorter than field populations.

They indicated that the bugs were more reluctant to feed on rabbit blood with less food intake and much longer development time.

Along the same lines, Montes et al. (2002) [23] used a Parafilm^®^ membrane to simulate the animal’s skin with different blood sources (cattle, sheep, chicken and lamb) for feeding bed bugs with an average colony-feeding rate of 90–100% with fresh heparinized blood. Egg production fell dramatically with commercial defibrinated blood that was unable to support a new generation.

*Feeding frequency* – This factor was strongly linked to temperature and host availability for bed bug populations [27]. A rate of two feedings per week seems to be closer to the naturally observed frequency [19, 26]. The majority of researchers fed their bed bug colonies weekly (67.8%), biweekly (16.9%), every three days (3.4%), continuously (3.4%) or did not report this information (8.5%).

*Life-cycle (egg-adult)* – Another major physiological factor for bug laboratory colonies is to verify complete life-cycle from egg to adult stages. This period is affected by the physical and physiological conditions. Any variation of the mentioned factors (mainly temperature and blood meal) affected the egg-adult time of different bed bugs (Table 1).

The bed bug life-cycle is usually short. *C. lectularius* and *C. hemipterus* attracted much attention to be colonized in the laboratory. At 28 °C, the *C. lectularius* life-cycle varied from 20 days feeding by rabbit blood to 23 days with human and rodent blood. Using an artificial feeder has become widespread since the early 2000 s.

## Discussion

The Cimicidae (*C. lectularius* and *C. hemipterus*) are all hematophagous with five nymphal stages. Bed bugs are normally domestic with natural habitats close to humans.

Worldwide distribution of these insects has been increasing with an impact on human health.

The dramatic worldwide recrudescence of bed bugs, coupled with chemical resistance, has fuelled research that focused mainly on control management, including the physical factors for their eradication. These factors, particularly temperature, were the subject of several publications that achieved these goals.

Bed bug infection has been found to have psychological effects in humans and has a major economic impact, particularly in regions with high tourism activity [6, 13, 14]. Recently, Salazar et al. (2014) [30] reported the vectorial competence of *C. lectularius* in laboratory experiments. *T. cruzi* is transmitted through faeces excreted directly on the host’s skin during the blood feeding. Scratching around the bite area is the main pathway for *Trypanosoma* penetration into the host.

Concerning the impact of Cimicidae on human health, improving our knowledge on their natural biology, ecological niches, hosts and environmental parameters is essential.

We found that different methodologies have been used by researchers to investigate these insects. Each research group used its own preferred method for experimental conditions based on accessibility, ease of use, etc. The available information allowed us to compare different colonies with preferred conditions at the bed bug species level.

Long-term laboratory colonization of bugs is believed to be multifactorial. Physical and physiological factors play important roles and any change in certain factors leads to critical variations, mainly in life-cycle. In addition, each species has its own preference for temperature and feeding conditions. As a result, researchers have long attempted to establish controlled laboratory conditions for these insects.

Some items have been considered as fundamental to a good and fertile colony. They include short nymph life-cycle and low mortality, long adult lifespan, high fecundity, fertility and high rate of hatching that are highly influenced by the factors we mentioned. Also moulting, mating, host location, activity and quiescence are all central life history elements that must be understood to combat the bugs.

*Physical factors* – The optimal physical factors for bed bugs are similar (Table 1).

High temperature can increase bug mortality before they reach the adult stage [28, 29] whereas it can accelerate the development cycle by accelerating bug metabolism. Therefore, considering the temperature threshold to accelerate and maintain bug colonies is essential.

Here, we concentrated on the role of temperature to facilitate bed bug breeding. Normally, the optimal temperature (26–28 °C) is suitable to rapidly obtain several generations. However, this temperature is beyond the usual comfort range for humans, i.e. 22–24 °C [4]. This comfort range in the studies targeting control management and human health seems to be an important baseline parameter and is potentially different from habitually used higher temperatures.

Besides temperature, relative humidity (RH) is another factor that influences bug populations in laboratory colonies. Some studies reported high impact of this factor in their colonies whereas others stated the opposite. Lower RH increases the necessity of bugs to take blood as a way of compensating for water loss [22]. Therefore, low RH would increase the feeding frequency in relation to dehydration and blood meals provide an important source of water [9].

The photoperiod is one of the environmental factors that has not been discussed sufficiently in studies performed on bug colonies. Most studies used light/dark cycles of 12 h/12 h for bed bug colonies. Therefore, the impact of this factor is somewhat ambiguous and requires further investigation to clarify its role on life-cycle and metabolism.

*Physiological factors* – Each bug species has its own preference for feeding conditions. An important factor with high impact on bug colonies is feeding with a suitable blood source.

Bug-host interactions are important in understanding nuisance phenomena and transmission of zoonotic diseases that can affect wildlife, domestic animals and human health. Therefore, knowledge of the bug’s natural host is an essential step in understanding the life-cycle. Some items, such as using the same natural host animal, or a different animal, and feeding by live animal or artificial feeder, are important items that affect colonies.

The Cimicidae have few natural host choices due to close living to human habitats. In the studies performed recently on bed bug colonies, half of them used artificial feeding, 23% used laboratory animals and 10% used humans as the blood source; 7% practised a combination of artificial and natural feeding and 10% did not describe their feeding method [1].

The recommendations of the European Convention for the Protection of Vertebrate Animals used for Experimental Purposes (CETS 123) clearly state that artificial feeders should be preferred to live animals, if possible.

It seems that, if possible, human blood should be used more often to understand its impact on the natural biology of bugs found in human habitats.

Food quality, including relative proportions of blood cells and plasma, may influence insect biology and affect bug colonies. Low blood viscosity will support a shorter period of bloodsucking, which may fail to elicit a behavioural response by the host [2].

Defibrinated blood is commonly used for bug colonies but it may lack certain nutrients normally acquired from a living host [3].

Human erythrocytes contain 0.136 g/100 mL of DNA compared with 4.216 g/100 mL in chickens.

Nucleic acid catabolism probably requires increased energy consumption that may also contribute to slowed development in bugs fed on pigeons [20], probably related to the nutritional characteristics of host blood [15].

There is a slight variation in egg production when insects feed from different host animals [17, 18].

In bug colonies fed fortnightly, larval development was faster with a lower mortality rate than those fed weekly, perhaps due to reduced bug handling [7].

Based on the results of De Meillon and Golberg (1946) [12], we observed different bed bug life-cycle durations on various living hosts, particularly between humans and guinea pigs (21%).

Bed bug life-cycle was much faster in comparison with Triatominae.

These observations lend support to the idea that the availability of nutritional resources can affect the bugs’ phenotype, physiology and behaviour.

Further studies are necessary to determine the possible relationships between adaptation to the intradomiciliary and laboratory environments.

## Conclusion

Concerning the factors we examined, we suggest that several items should be taken into account to establish and maintain bug colonies. Firstly, bug biology at the species level and its geographic localization are important. Secondly, applying optimal physical and physiological conditions increases laboratory bug populations. Temperature has a major impact on life-cycle duration, while the type of blood meal influences the quality of biological functions, for instance fertility and fecundity.

When possible, or depending on the aim of the studies, to standardize bed bug colonies, it would be beneficial to use natural conditions: 22–24 °C, 30–50% RH with artificial feeding using human blood. Indeed, there are only two species with very similar and stable habitats and human hosts.
